# Prevalence of people at risk of developing type 2 diabetes mellitus and the involvement of community pharmacies in a national screening campaign: a pioneer action in Brazil

**DOI:** 10.1186/s13098-020-00593-5

**Published:** 2020-10-08

**Authors:** Cassyano J. Correr, Wendel Coura-Vital, Josélia C. Q. P. Frade, Renata C. R. M. Nascimento, Lúbia G. Nascimento, Eliete B. Pinheiro, Wesley M. Ferreira, Janice S. Reis, Karla F. S. Melo, Roberto Pontarolo, Mônica S. A. Lenzi, José V. Almeida, Hermelinda C. Pedrosa, Walter S. J. João

**Affiliations:** 1 Departamento de Farmácia, Universidade Federal do Paraná, Curitiba, Paraná Brazil; 2 Programa de Pós Graduação em Ciências Farmacêuticas, Escola de Farmácia, Universidade Federal de Ouro Preto, Ouro Preto, Minas Gerais Brazil; 3 Conselho Federal de Farmácia, Brasília, Distrito Federal Brazil; 4 Sociedade Brasileira de Diabetes, Ensino e Pesquisa da Santa Casa de Belo Horizonte, Belo Horizonte, Minas Gerais Brazil; 5 Sociedade Brasileira de Diabetes, Equipe de Diabetes do Hospital das Clínicas da Faculdade de Medicina da Universidade de São Paulo, São Paulo, Brazil; 6 Sociedade Brasileira de Diabetes, São Paulo, Brazil; 7 Secretaria de Estado da Saúde, Polo de Pesquisa da Unidade de Endocrinologia FEPECS-HRT, Brasília, Distrito Federal Brazil

**Keywords:** Diabetes *mellitus*, Risk factors, Chronic disease, Prevalence

## Abstract

**Background:**

Brazil is one of top 10 countries with the highest number of people with diabetes mellitus (DM), affecting 16.8 million peoples. It is estimated that 7.7 million people (20–79 years) in the country have not yet been diagnosed, representing an under-diagnosis rate of 46.0%. Herein we aimed to screen people for high blood glucose or risk for developing type 2 DM (T2DM) through community pharmacies in Brazil.

**Methods:**

A cross-sectional study was carried out in November 2018, involving 977 pharmacists from 345 municipalities in Brazil. The study evaluated people between 20 and 79 years old without a previous diagnosis of DM. Glycemia was considered high when its value was ≥ 100 mg/dL fasting and ≥ 140 mg/dL in a casual feeding state. The FINDRISC (Finnish Diabetes Risk Score) was used to estimate the risk for developing T2DM. The prevalence of high blood glucose was estimated and the associated factors were obtained using Poisson's multivariate analysis with robust variance.

**Results:**

During the national screening campaign, 17,580 people were tested with the majority of the consultations (78.2%) being carried out in private pharmacies. The population was composed mainly of women (59.5%) and people aged between 20 and 45 years (47.9%). The frequency of participants with high blood glucose was 18.4% (95% CI 17.9–19.0). Considering the FINDRISC, 22.7% of people had a high or very high risk for T2DM. The risk factors associated with high blood glucose were: Body Mass Index > 25 kg/m^2^, abdominal circumference > 94 cm for men and > 80 cm for women; education level below 15 years of study, no daily intake of vegetables and fruits; previous diagnosis of arterial hypertension; history of high blood glucose and family history of DM.

**Conclusions:**

This is the largest screening study that evaluated the frequency of high blood glucose and its associated factors in a population without a previous diagnosis ever performed in community pharmacies in Brazil. These results may help to improve public health policies and reinforce the role of pharmacists in screening and education actions aimed at this undiagnosed population in a continent-size country such as Brazil.

## Background

The epidemiological transition is a major challenge for health systems and societies in the Americas [1]. Population aging, urbanization, and changes in lifestyle have impacted the prevalence of chronic noncommunicable diseases (NCD), with emphasis on hypertension, diabetes mellitus, and dyslipidemia [1, 2].

Brazil is one of the top 10 countries in the world with the highest number of people with DM and currently is the 5th in the ranking [3]. International estimates indicate a growing trend in the prevalence of DM in the country, with an expected increase ranging from 6 to 7.8% in 2030 [4]. Despite the lack of nationwide studies on the prevalence of DM, results from the Vigitel 2017 household survey showed a prevalence of 7.7% in the 27 Brazilian capitals, with 7.1% for men and 8.1% for women [5]. The 2019 9th IDF Diabetes Atlas estimated that in in the Brazilian population between 20 and 79 years old there are approximately 16.8 million people with diabetes in Brazil, and 7.7 million undiagnosed DM, representing an under-diagnosis proportion of 46.0% [3].

Several studies have pointed out impressive data on pre-diabetes in Brazil. The National Health Survey, conducted from 2014–2015, which used glycated hemoglobin (HbA1c) as a diagnostic tool in a sample over 8,500 people, identified a prevalence of pre-diabetes ranging from 6.8% to 16.9%, depending on the criteria used [6]. The ELSA longitudinal study, involving Brazilian adults, reported pre-diabetes prevalence ranging from 20–59% of the sample recruited among university professional staff [7]. In addition to the diagnostic challenges, control rates of DM in Brazil remain unsatisfactory. The last largest nationwide study analyzing glucose control was conducted in 2006 and showed that 75% of 6.671 individuals with T2DM and 90% of those with type 1 DM (T1DM) assisted in private and public services by either specialists or no-specialists presented HbA1c higher than 7% [8]. Other studies have shown similar or even worse results among T1DM [9, 10].

In Brazil, the treatment of diabetes micro and macrovascular complications represents the main component of the disease expenses, with 48.2% of the cost attributed to medicines to treat these complications [11, 12]. Therefore, early diagnosis of T2DM is important to prevent chronic complications associated with the disease and to reduce the costs associated with health care delivery.

The Finnish Diabetes Risk Score (FINDRISC) is a simple, non-invasive and easy-to-use screening instrument used to predict the risk of an adult developing DM2 in 10 years [13]. This instrument has been adopted worldwide to lead T2DM prevention programs in primary health care [14–17] and has been adapted by the Brazilian Diabetes Society to be applied during national awareness campaigns in Brazil [18]. Studies conducted in several countries have shown that pharmacies are successful partners in programs for screening and diagnosing diabetes and in the identification of risk factors for cardiovascular diseases [19–23]. In Brazil, pharmacies are establishments that provide pharmaceutical services as well as individual and collective health guidance, including assessment of capillary blood glucose, blood pressure measurement, among other procedures. Pharmacists are higher education health professionals with responsibilities for carrying out clinical activities, including screening, education, and monitoring of patients [24, 25]. In this context, the present study aimed to identify, among people without a previous diagnosis of DM, the frequency of high blood glucose in the Brazilian population and the associated factors.

## Methods

### Study design and population

A cross-sectional study was carried out in November 2018, involving 977 pharmacists from 345 municipalities in Brazil, to assess the frequency of high blood glucose in individuals without a previous diagnosis of DM.

Brazil is the largest country in South America, with a territorial area of 8,510,820.623 Km^2^, divided into five geographic regions (North, South, Midwest, Southeast, and Northeast) and subdivided into 27 federative units, including the Federal District. The estimated national population, for the year 2018, was 210,867,954 inhabitants [26]. To calculate the ideal sample size, there were considered the Brazilian population aged between 20 and 79 years (141,802,185 people; IBGE 2017); an estimated prevalence of pre-diabetes or DM of 23% [27]; 95% confidence level; and 1% estimate accuracy. The estimated sample for the country (11,750) was further stratified taking into account the population aged 20 to 79 years in each federative unit.

People aged between 20 and 79 years old who were attended at any of the selected pharmacies, during the study period, were invited by pharmacists to participate in the study. Participants could be in their feeding routine (unknown diet) or fasting (8 h without any caloric intake). The exclusion criteria were: participants’ records presenting blood glucose data collected with glucometers without ISO 15197: 2013 certification (ISO, 2013); previous diagnosis of DM; any data collected with non-standard anthropometric tape; age < 20 years or > 79 years; lack of data on weight (measured on a scale), height, Body Mass Index (BMI) and or abdominal circumference (AC); capillary blood glucose (CBG) lower than 70 mg/dL without confirmation or CBG higher than 300 mg/dL without confirmation.

### Selection of pharmacies and training of pharmacists

Participated in the study volunteer pharmacists from community pharmacies (private and public), located in different municipalities throughout the national territory. Community pharmacy is a type of health care facility that provides pharmaceutical services or with a given mission around medicines [24, 28]. Pharmacies were selected in a non-probabilistic manner, by fulfilling the following criteria: (i) ability to collect and send data by electronic means; (ii) agreeing to use their inputs (glucometer, blood glucose test strips, scale, anthropometric tape, office supplies); (iii) be located in a municipality that has a public primary health care service and that was covered by the Mobile Emergency Care Service; (iv) be regularly enrolled in the Regional Pharmacy Council of their jurisdiction, with full-time pharmaceutical coverage; (v) have a patient care room allowing visual and sound privacy (pharmaceutical care room).

The recruitment of the the study population was carried out by broadcasting media campaigns (television, radio, Federal Pharmacy Board website [29], and a website developed exclusively for the campaign [30]) and locally, by the participating pharmacies. Standardized disclosure materials were provided to participating pharmacies.

### Testing protocol and data collection

During November 2018, people who attended any of the selected pharmacies were invited by the pharmacist to participate in the study, being informed about the objectives of the project and the inclusion criteria. Those who agreed to participate signed the Free and Informed Consent Form, answered a questionnaire with personal information, clinical data and the FINDRISC [13], translated and cross-culturally adapted to Brazilian Portuguese [31]. The FINDRISC analyses eight clinical characteristics: age, BMI, AC, daily physical activity, eating habits, blood pressure, use of antihypertensive medication, history of hyperglycemia, and family history of diabetes. The final score corresponds to the sum of the scores attributed to each question, ranging from 0 to 26, and the individual risk of developing T2DM was stratified into five categories, ranging from low to very high risk [15]. Subsequently, anthropometric measurements and CBG test were performed. Height was obtained with a tape measure fixed on the wall. Weight was obtained by a digital or analog scale registered by the National Health Surveillance Agency (Anvisa), available at the time of the evaluation. BMI was obtained by dividing the weight (in kilograms) by the square of height (in meters); AC was measured using a professional anthropometric tape, following the recommendations of the Brazilian Association for the Study of Obesity and Metabolic Syndrome [32]; CBG tests were performed using glucometers registered by Anvisa, with ISO 15,197: 2013 (ISO, 2013) certification, such as Accu Check Performa® (Roche), Accu Check Performa Connect® (Roche), Accu Check Guide® (Roche), One Touch Select Plus Flex® (J&J), Contour Plus® (Bayer), G-Tech Free Lite (GTech), Freestyle Freedom Lite® (Abbott), and Freestyle Optium Neo® (Abbott). The results were interpreted according to the recommendations of the Brazilian Diabetes Society, considering the patient's feeding status at the time of the CBG test [18]. CBG levels less than 100 mg/dL were considered normal when fasting or less than 140 mg/dL, in a casual feeding state.

The interpretation of the CBG result and the subsequent orientation to the participant were performed by the pharmacist. Participants with results suggestive of pre-diabetes or DM were referred to the local public or private health system, with a proper filled in declaration form [25], containing the result of the parameters evaluated during the screening procedures.

### Data analysis

After filling in the survey instruments, the data were entered into an online platform (Surveymonkey) and later exported for analysis using the Stata Version 14.0 software.

A difference observed in the degrees of risk defined by FINDRISC and CBG levels were evaluated by chi-square test.

The Poisson multivariate model, with robust variance, was used to identify the risk factors associated with high blood glucose and the estimates were obtained using the prevalence ratio (PR) with 95% CI. Initially, a univariate analysis was performed and the variables that showed a statistical association in this analysis (*p* < 0.25) were analyzed in a multivariate model. Variables with more than two categories were transformed into dummy variables. For the construction of the final model, a complete model was started, containing all the variables, and the successive disposal of the variables was carried out until only those with significance level remained in the model (*p* < 0.05).

## Results

**Fig. 1:**
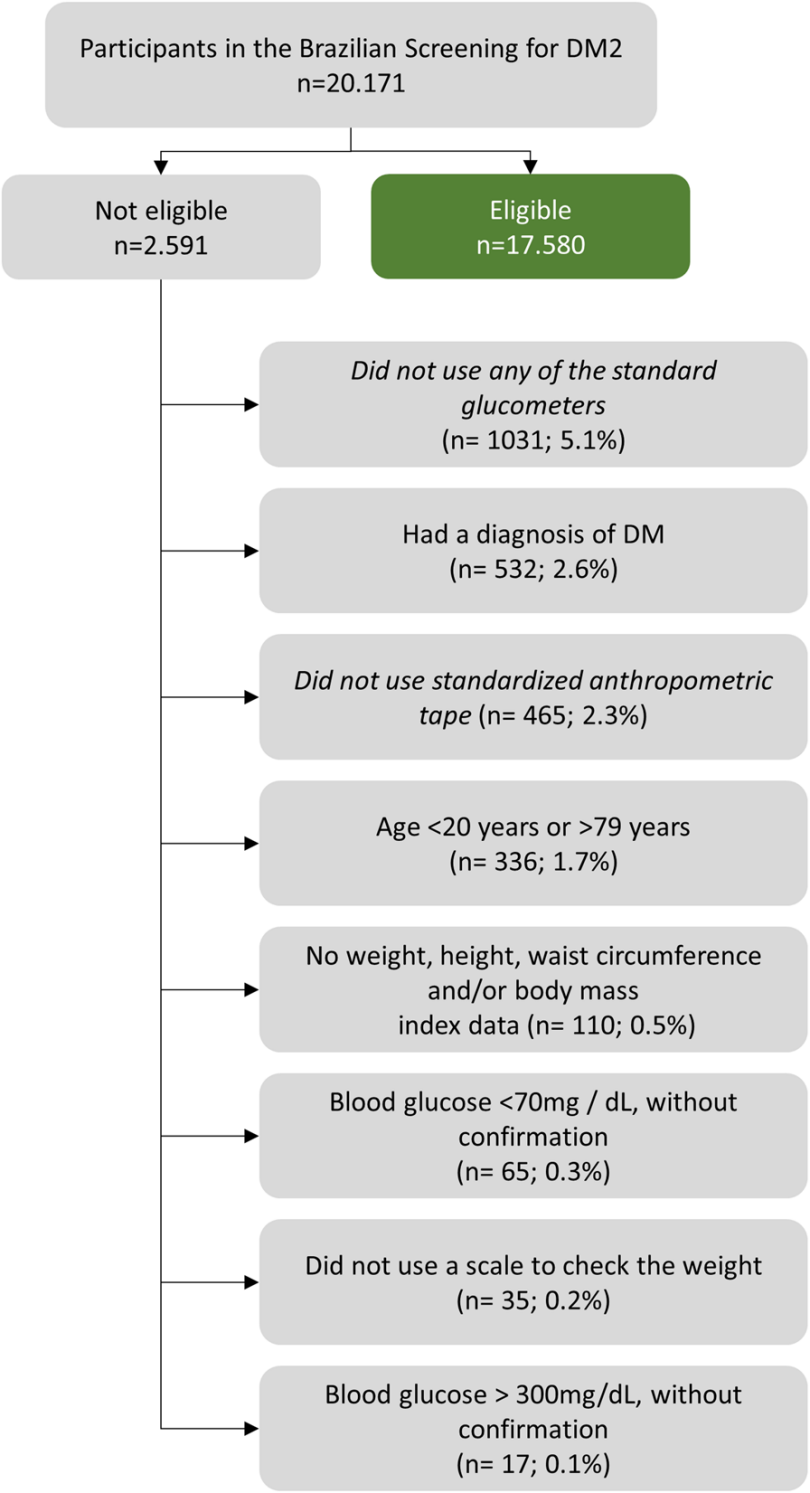
Population included in the screening of high blood glucose in Brazil, 2018

The final sample screened was 20,171 participants, of which 17,580 (87.2%) were eligible for the study (Fig. 1).

### Characteristics of the assessed population

Of the 17,580 participants included, most were women (59.5%) and the predominant age group was less than 45 years old (47.9%). The mean and median age were 46 ± 15.7 years, with the first and third quartiles being 33 and 59, respectively. The predominant skin color was white (47.3%), followed by brown (38.0%). In terms of education, 77.7% of the participants had more than 8 years of study, 18.0% reported 8 to 10 years of study, 29.1% indicated 11 to 14 years, and 30.6% informed 15 or more years of study. Regarding the practice of physical activities, 68.3% reported not practicing any activity and, concerning eating habits, 43.0% did not eat vegetables or fruits daily. It was observed that 30.8% of the sampled population self-reported hypertension or used antihypertensive medication. As for blood glucose rates, 15.8% reported having high glucose levels events in the past and 58% informed a family history of T1DM or T2DM (Table 1).

**Table 1:** Baseline characteristics of the population included in the screening of high blood glucose in Brazil, 2018

Variables	n	%
Gender		
Male	7112	40.5
Female	10,468	59.5
Age (years)		
20–45	8412	47.9
45 − 54	3374	19.2
55 − 64	3188	18.1
65–79	2606	14.8
Abdominal circumference (cm)		
Male < 94	2916	41.0
94 − 102	2099	29.5
> 102	2097	29.5
Female < 80	2222	21.2
80 − 88	2184	20.9
> 88	6062	57.9
Color		
White	8318	47.3
Brown	6679	38.0
Black	2253	12.8
Yellow	192	1.1
Indigenous	52	0.3
Uninformed	86	0.5
Educational level (years)		
Illiterate	814	4.6
1 − 3	173	1.0
4 − 7	2936	16.7
8 − 10	3166	18.0
11 − 14	5108	29.1
≥ 15	5383	30.6
Practice of physical activity		
Yes	5575	31.7
No	12,005	68.3
Consumption of vegetables and/or fruits		
Every day	10,024	57.0
Do not eat every day	7556	43.0
Diagnosis or use of medicines for hypertension		
Yes	5413	30.8
No	12,167	69.2
High blood glucose in the past		
Yes	2780	15.8
No	14,800	84.2
Family member with type 1 or 2 diabetes mellitus		
No	7377	42.0
Yes (grandparents, uncles, cousins)	3741	21.3
Yes: parents. siblings or children	6462	36.7

### Risk of developing DM2 in the next 10 years

According to the FINDRISC, 22.1% (n = 3873) were at low risk for developing T2DM (1 in 100 develops the disease); 35.1% (n = 6169) slightly moderate risk (1 in 25 develops the disease); 20.1% (n = 3523) moderate risk (1 in 6 develops the disease); 19.6% (n = 3436) high risk (1 in 3 develops the disease) and 3.1% (n = 555) very high risk (1 in 2 develops the disease) (Table 2).

**Table 2:** Risk for developing T2DM (FINDRISC), according to capillary blood glucose levels, regardless of dietary status, Brazil, 2018

FINDRISC	Blood glucose (mg/dL)*		Total n (%)
	Normal n (%)	High n (%)	
Low risk	3565 (24.9)	308 (9.5)	3873 (22.1)
Slightly moderate risk	5275 (36.8)	894 (27.6)	6169 (35.1)
Moderate risk	2828 (19.8)	695 (21.4)	3523 (20.1)
High risk	2350 (16.4)	1086 (33.5)	3436 (19.6)
Higher risk	297 (2.1)	258 (8.0)	555 (3.1)

*Glycemia considered normal: fasting < 100 mg/dL or casual < 140 mg/dL. Results excluding 24 patients with blood glucose results < 70 mg/dL

Among the participants with low or moderate risk, 24.9 and 36.8%, respectively, had normal CBG. Participants who presented high or very high FINDRISC were mainly those who had high CBG levels, with percentages much higher than those found in individuals with normal CBG, corresponding to 33.5 and 8.0% respectively.

### Frequency of high capillary blood glucose levels

**Fig. 2:**
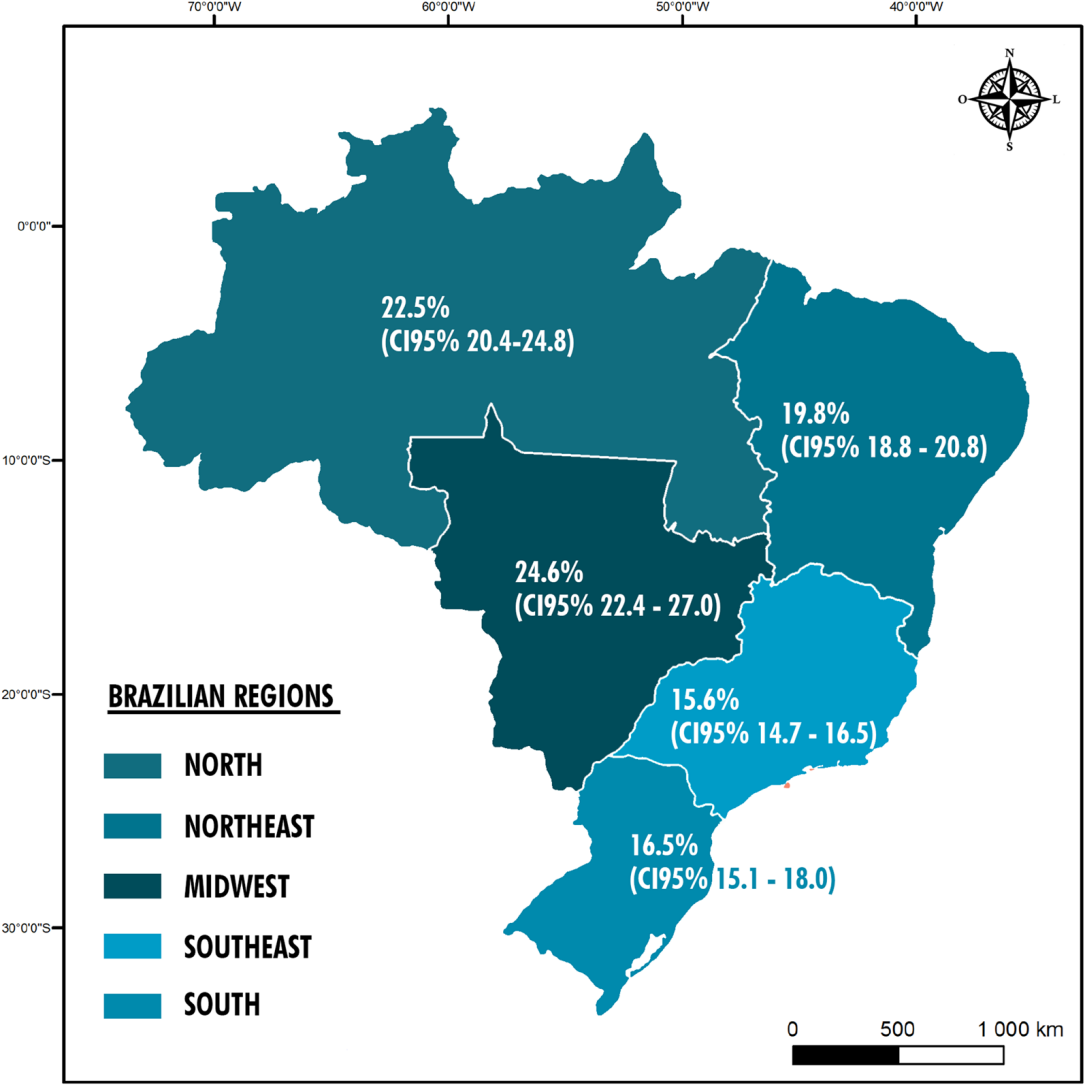
Prevalence of high capillary blood glucose, according to Brazilian region, 2018

The frequency of high CBG levels (fasting CBG ≥ 100 mg/dL or in random condition ≥ 140 mg/dL) among the individuals enrolled in this present screening was 18.4% (95% CI 17.9–19.0). When the frequency by geographic region was assessed, the Midwest presented the highest frequency (24.6%; 95% CI: 22.4 − 27.0), followed by the North (22.5%; 95% CI 20.4 − 24.8) and Northeast (19.8%; 95% CI: 18.8 − 20.8) regions (Fig. 2).

### Risk factors for high capillary blood glucose

Preliminary selection of variables (*p* < 0.25), through univariate analysis, is described in Table 3. According to multivariate analysis (Table 4), the BMI of 25–30 kg/m^2^ (PR 1.1) and BMI > 30 kg/m^2^ (PR 1.3), when compared with BMI < 25 kg/m^2^; AC for men of 94–102 cm or for women of 80–88 cm (PR 1.2) as well as AC for men > 102 cm or for women > 88 cm (PR 1.3) compared to AC < 94 cm for men or < 80 cm for women, increased the risk of high CBG. People with less education had a higher frequency of high CBG, compared to people with 15 or more years of education (illiterate PR 1.5). People who did not eat fruits or vegetables every day (PR 1.1), reported being hypertensive or use medicines for high blood pressure (PR 1.1), and those informing a high glycemia rate in the past (PR 1.9) and having first-degree relatives with either T1DM or T2DM (PR 1.3) were associated with a higher prevalence of high CBG. The PR values obtained in the multivariate model were adjusted for the participants' age and sex.

**Table 3:** Univariate analysis of population characteristics associated with high blood glucose levels, Brazil, 2018

Variables	Blood glucose (mg/dL)*		PR (CI95%)	*p* value
	Normal (n/%)	High (n/%)		
Gender				
Male	8720 (60.9)	1734 (53.5)		
Female	5595 (39.1)	1507 (46.5)	1.3 (1.2 − 1.4)	0.01
Age (years)				
20–45	7486 (52.3)	913 (29.2)		
45–54	2655 (18.5)	716 (22.1)	1.9 (1.8 − 2.1)	0.01
55–64	2357 (16.5)	827 (25.5)	2.4 (2.2 − 2.6)	0.01
65–79	1817 (12.7)	785 (24.2)	2.8 (2.6 − 3.0)	0.01
Body mass index				
< 25	5220 (35.5)	782 (24.1)		
25–30	5595 (39.1)	1326 (40.9)	1.5 (1.4 − 1.6)	0.01
> 30	3500 (24.5)	1133 (35.0)	1.9 (1.7 − 2.0)	0.01
Abdominal circumference (cm)				
♂ < 94 or ♀ < 80	4491 (31.4)	635 (19.6)		
♂ 94–102 or ♀ 80–88	3518 (24.6)	760 (23.4)	1.4 (1.3 − 1.6)	0.01
♂ > 102 or ♀ > 88	6306 (44.0)	1846 (57.0)	1.8 (1.7 − 2.0)	0.01
Color				
White	6821 (47.6)	1489 (45.9)		
Brown	5471 (38.2)	1200 (37.0)	1.0 (0.9 − 1.1)	0.91
Black	156 (1.1)	36 (1.1)	1.0 (0.8 − 1.4)	0.77
Yellow	1754 (12.3)	491 (15.2)	1.2 (1.1 − 1.3)	0.01
Indigenous	44 (0.3)	8 (0.3)	0.8 (0.4 − 1.6)	0.64
Uninformed	69 (0.5)	17 (0.5)	1.1 (0.7 − 1.7)	0.65
Educational level (years)				
≥ 15	4644 (32.4)	734 (22.7)		
11 − 14	4347 (30.4)	752 (23.2)	1.1 (1.0 − 1.2)	0.10
8 − 10	2498 (17.4)	665 (20.5)	1.5 (1.4 − 1.7)	0.01
4 − 7	2143 (15.0)	791 (24.4)	2.3 (2.1 − 2.6)	0.01
1 − 3	123 (0.9)	49 (1.5)	2.5(1.8 − 3.5)	0.01
Illiterate	560 (3.9)	250 (7.7)	2.8 (2.4 − 3.3)	0.01
Practice of physical activity				
Yes	4608 (32.2)	964 (29.7)		
No	9707 (67.8)	2277 (70.3)	1.1 (1.0 − 1.2)	0.01
Consumption of vegetables and/or fruits				
Every day	8216 (57.4)	1796 (55.4)		
Do not eat every day	6099 (42.6)	1445 (44.6)	1.1 (1.0 − 1.1)	0.04
Diagnosis or use of medicines for hypertension				
No	10,362 (72.4)	1787 (55.1)		
Yes	3953 (27.6)	1454 (44.9)	1.8 (1.7 − 1.9)	0.01
High blood glucose in the past				
No	12,533 (87.5)	2243 (69.2)		
Yes	1782 (12.5)	998 (30.8)	2.4 (2.2 − 2.5)	0.01
Family member with type 1 or 2 diabetes mellitus				
No	6159 (43.0)	1204 (37.1)		
Yes (grandparents, uncles, cousins)	3228 (22.5)	505 (15.6)	0.8 (0.7 − 0.9)	0.01
Yes: (parents, siblings or children)	4928 (34.4)	1532 (47.3)	1.4 (1.3 − 1.5)	0.01

*Glycemia considered normal. fasting < 100 mg/dL or casual < 140 mg/dL. Results excluding 24 patients with blood glucose results < 70 mg/dL. ♂—Male; ♀—Female

**Table 4:** Multivariate analysis of characteristics associated with high blood glucose, Brazil, 2018

Variables	PR (CI95%) crude	PR (CI95%) ajusted*
Body mass index		
< 25	−	−
25–30	1.5 (1.4 − 1.6)	1.1 (1.1 − 1.2)
> 30	1.9 (1.7 − 2.0)	1.3 (1.2 − 1.4)
Abdominal circumference (cm)		
♂ < 94 or ♀ < 80	−	−
♂ 94–102 or ♀ 80–88	1.4 (1.3 − 1.6)	1.2 (1.1 − 1.3)
♂ > 102 or ♀ > 88	1.8 (1.7 − 2.0)	1.3 (1.2 − 1.5)
Educational level (years)		
≥ 15	−	−
8 − 10	1.5 (1.4 − 1.7)	1.3 (1.1 − 1.4)
4 − 7	2.3 (2.1 − 2.6)	1.4 (1.3 − 1.5)
1 − 3	2.5 (1.8 − 3.5)	1.5 (1.2 − 1.9)
Illiterate	2.8 (2.4 − 3.3)	1.5 (1.3 − 1.7)
Consumption of vegetables and/or fruits		
Every day	−	−
Do not eat every day	1.1 (1.0 − 1.1)	1.1 (1.1 − 1.2)
Diagnosis or use of medicines for hypertension		
No	−	−
Yes	1.8 (1.7 − 1.9)	1.1 (1.1 − 1.2)
High blood glucose in the past		
No		
es	2.4 (2.2 − 2.5)	1.9 (1.8 − 2.0)
Family member with type 1 or 2 diabetes mellitus		
No	−	−
Y es (parents, siblings or children)	1.4 (1.3 − 1.5)	1.3 (1.2 − 1.4)

♂—Male; ♀—Female. *Final model adjusted for age and sex

## Discussion

This is the largest study ever conducted in pharmacies in all regions of Brazil that assessed the frequency of high blood glucose levels and the risk of developing diabetes. Performing NCD screening is important because it can identify asymptomatic people at high risk or with initial signs and symptoms of a disease, allowing for timely diagnosis and treatment, which is very important for a disease such as diabetes. According to the WHO, a substantial fraction of the disease burden and mortality due to NCD are related to a small set of risk factors, among which stand out smoking, inadequate food consumption, physical inactivity, and excessive consumption of alcoholic beverages [33]. Early detection of people at risk of developing T2DM is very important, since changes in lifestyle, including physical activity and proper diet, can reduce the incidence of the disease by approximately 58% [14, 18]. In addition, its early detection and intervention in patients with T2DM can minimize complications from other comorbidities such as non-alcoholic fatty liver disease, with insulin resistance being a common pathophysiological mechanism that mutually interferes in the progression of both diseases [34, 35].

It was found that the frequency of high CBG levels was higher in the Midwest, North, and Northeast regions. These findings can be explained by the heterogeneous profile of the Brazilian population, related to lifestyle and prevalence of obesity which has been increasing. In Brazil, overweight (BMI > 25 kg/m^2^) and obesity rates are spreading in all age groups, both sexes, and all income levels, with the obesity rates being more frequent in the population with lower family income [36]. Indeed, according to data from Vigitel 2018 [37], the prevalence of obesity (BMI > 30 kg/m^2^) in adults is 19.8%, with the highest frequencies observed in capitals of the Midwest, North, and Northeast regions, with emphasis on the capital cities like Cuiabá (23.0% CI 95% 20.5–25.4), Manaus (23.0% CI 95% 19.7–26.3), and Recife (21.9% CI 95% 19.3–24.4).

Regarding the consumption of fruits and vegetables, a similar distribution was found, with the lowest percentages observed in the North and Northeast regions [37]. The Vigitel survey also pointed out that consumption of fruits and vegetables is more frequent among women and, despite the increase in the consumption of these foods observed in the last 10 years, only 23.1% of Brazilians consume the 5 daily portions at least 5 times a week, as recommended by the WHO [37]. Generally, diets have a high sugar intake, acting as the main source of advanced glycation endproducts (AGEs). These compounds are toxic and are associated with the pathogenesis of diet-related diseases, such as diabetes, insulin resistance, among others [38]. The AGEs, due to hyperglycemia and insulin resistance present in people with diabetes, are an extra source of reactive oxygen species (ROS) which overload the cellular antioxidant machinery, leading the cell to a state of oxidative stress [39]. Thus, natural compounds with antioxidant properties, such as citrus fruits, tomatoes, green tea, can prevent the harmful effects of AGEs, providing benefits in the control of T2DM.[38, 40]. In turn, ROS mediate chronic inflammatory processes that are directly related to the micro and macrovascular complications of diabetes, which are diabetic retinopathy, neuropathy and nephropathy [41, 42]. Inflammatory processes can also be triggered by diets with low intake of vitamin D, a fact corroborated by study that show an inverse correlation between blood levels of vitamin D and C-reactive protein. This relationship being more pronounced in patients with inflammatory diseases, such as diabetes, compared to patients with non-inflammatory diseases [43]. In addition, it has been speculated that vitamin D supplementation may contribute to reducing the risk of developing diabetes in patients at risk of developing the disease [44].

Similar to the results reported by Passos et al. (2005), it was verified a positive relationship between physical inactivity and high CBG [45]. Sedentary lifestyle has been associated with insulin resistance in individuals without a diagnosis of diabetes, regardless of obesity [46, 47]. Reporting of previous arterial hypertension was also associated with high CBG. It is known that hypertension has a prevalence up to three times higher in people with DM when compared to those who do not have the disease, being the major determinant of atherosclerotic cardiovascular diseases (ASCVD) in this population [48, 49].

The percentage of people at high and very high risk of developing T2DM in Brazil was similar to the screening performed in pharmacies in Spain (23.5% had a high risk of diabetes -FINDRISC > 14 points) [50] and smaller than the risk reported by the screening performed in Thailand, in seven pharmacies, which showed a prevalence of 48% of high risk for DM [51]. The frequency of high CBG in Brazil was higher than the prevalence found in other countries such as Spain, Switzerland, and Thailand [50–52], indicating the need for multidisciplinary monitoring of these people.

T2DM is characterized by a long asymptomatic period before the diagnosis [14]. The early diagnosis of T2DM contributes to the reduction of ASCVD, prevention of microvascular complications (retinopathy, neuropathy, kidney disease), and premature mortality [14, 47, 53, 54]. People with T2DM have a two to four times greater risk of developing coronary heart disease, when compared to the general population, and approximately 8% of people with pre-diabetes develop retinopathy [47]. Moreover, more recent data have shown high percentages of peripheral neuropathy among people with obesity and pre-DM (29% versus 11% with normal glucose), and ranges from 11 to 34% among individuals with pre-DM [55]. Thus, early interventions to prevent or delay progression to T2DM represent an important benefit not only for increasing life expectancy and quality of life, but also reducing costs related to the management of the disease and its complications [15].

Success in the treatment of DM depends on the concomitant implementation of three categories of interventions: educational, self-monitoring, and pharmacological strategies. Whenever possible, it is recommended that care for people with diabetes should include a health care professional (HCP) interdisciplinary team composed of professionals with the proper qualification and practical experience in health education activities [56]. Pharmacists working in community pharmacies can contribute not only with HCP screening and education, but also with the monitoring of patients with DM, in collaboration with other HCP thus helping to improve the treatment and disease control.

A systematic review and meta-analysis assessing the impact of pharmaceutical care for people with diabetes, in outpatient services, revealed a 1.1% reduction in HbA1c compared to standard care (95% CI 0.88–1.27) [57]. In Brazil, a 12 months study enrolling patients with DM, followed in a pharmacy, showed a reduction of 2.2% in HbA1c compared to 0.3% in the control group [58]. These studies suggest that the health care intervention by the pharmacist in patients with DM can improve the HbA1c results acting as an “additional” effect to the standard care provided to the patient. Other parameters such as blood pressure, LDL cholesterol, triglycerides, BMI, and coronary risk were also sensitive to pharmaceutical intervention in this group of patients, as reported by Pousinho et al. [59].

Barcelo et al. [60] found that most of the direct costs of treating DM in Latin America are related to the treatment of complications. Additionally,the cost of a patient without chronic complications with HbA1c > 10% is 2.4 times higher when compared to a better controlled patient, with HbA1c < 8%. If the patient develops chronic complications, the resultant cost is 34 times higher [61]. Therefore, population-based DM screening actions can generate cost-effective results [62].

The campaign to track suspected cases of DM in Brazil, in 2018, reached a population larger than the number initially estimated, reinforcing the role of pharmacies in health promotion and disease prevention activities. As in other countries [51, 52], pharmacies in Brazil are health establishments that provide pharmaceutical services, individual and collective health guidance, handling and/or dispensing medications, and evaluation of CBG, blood pressure and weight measurements, among other procedures [24, 25]. Thus, pharmacies are favorable environments for carrying out clinical activities, including screening, education, and monitoring of people with NCD, particularly diabetes [63].

## Strengths and limitations

This is the first Brazilian T2DM screening study in pharmacies,using CBG test. The sample screened in this study was higher than expected; thus, the approach presented herein reinforces the strategic importance of the health services provided by community pharmacies for screening actions across the country. Another strength of the study was the opportunity to expand the application of the FINDRISC tool, which has been already translated and validated for the Brazilian Portuguese and is strongly recommended by the Brazilian Diabetes Society in its yearly detection campaigns. The completion of the questionnaire by health professionals, as performed herein, is expected to have increased the reliability of the data [14].

This study has some limitations. Because it is a cross-sectional study, a causal relationship cannot be established. Another possible limitation was the population screened since it was a convenience sample and most participants had 11 or more years of formal education plus the fact that the majority of the pharmacies were private. Therefore, it is possible that the more vulnerable and least assisted people and also frequently less educated were under evaluated. In Brazil, 20.6% of the inhabitants have an educational level of less than 3 years of formal education [64] and only 5.6% of the evaluated sample had this characteristic. Thus, although the frequency reported herein is alarming, it may be underestimated.

## Conclusions

This is the largest study ever conducted in Brazil to assess the frequency of high blood glucose levels and the risk for the development of T2DM, performed by pharmacists,. The profile of the people who were found to have high CBG indicates that strategies involving health education measures, encouragement to healthy eating, physical activity and weight loss could help to reduce the prevalence of T2DM in the country, especially in the most underserved regions of the country, North, Northeast and Midwest where the increase in obesity is an important concern. Therefore, pharmacists working in community pharmacies may contribute to screening of NCD, such as T2DM, and perform health education, in the early referral of suspected cases to the health service for diagnostic confirmation, as well as in the monitoring of the patient with DM, in collaboration with the other health professionals and produce better results in the disease control.

Public health actions directed to the population with high CBG can enable the early diagnosis of T2DM, and contributes to the reduction of ASCVD, prevention of microvascular complications and avoid premature mortality. Worth to quote the partnership between the Brazilian Diabetes Society and the Federal Council of Pharmacists which represents an important step towards the liaison of pharmacists and other HCP involved in screening and care of people with diabetes in Brazil.

## Data Availability

The datasets used during the current study are available from the corresponding author on reasonable request.

## Abbreviations

ASCVD: Atherosclerotic Cardiovascular Diseases
BMI: Body Mass Index
CBG: Capilar Blood Glucose
CI: Confidence Interval
DM: Diabetes Mellitus
FINDRISC: Finnish Diabetes Risk Score
HCP: Health Care Professional
NCD: Non-Communicable Disease
PR: Prevalence ratio
T1DM: Type 1 Diabetes Mellitus
T2DM: Type 2 Diabetes Mellitus
